# Impact of postoperative pulmonary complications on clinical outcomes in liver transplantation: a single-center retrospective analysis

**DOI:** 10.1186/s12890-026-04328-6

**Published:** 2026-05-11

**Authors:** Gürkan Değirmencioğlu, Deniz Kütük, M. Hanifi Çanakcı, Gülnur Kul, Serap Akçalı Duru, Şener Balas

**Affiliations:** 1 Department of General Surgery, Ankara Etlik City Hospital, Ankara, Turkey; 2 Department of Infectious Diseases, Ankara Etlik City Hospital, Ankara, Turkey; 3 Department of Chest Diseases, Ankara Etlik City Hospital, Ankara, Turkey

**Keywords:** Liver transplantation, Postoperative pulmonary complications, Atelectasis, Intensive care unit stay, Mortality

## Abstract

**Background:**

Liver transplantation (LT) is the definitive treatment for end-stage liver disease. However, postoperative pulmonary complications (PPCs) significantly contribute to morbidity and mortality. This study aims to analyze the incidence, risk factors, and clinical impact of PPCs in adult LT recipients.

**Methods:**

We retrospectively analyzed 86 adult patients who underwent LT at our tertiary referral center. Patients were categorized based on the development of PPCs, including pleural effusion, atelectasis, ARDS, pneumonia, and the need for reintubation. Demographic data, perioperative variables, and clinical outcomes (ICU stay, hospital stay, and 30-day mortality) were compared.

**Results:**

The overall incidence of postoperative pulmonary complications (PPCs) was 46.5%. Atelectasis (32.6%) and reintubation (22.1%) were the most frequent complications. Patients who developed PPCs had significantly longer ICU stays (11.0 vs. 4.0 days, *p* < 0.001) and hospital stays (19.5 vs. 16.5 days, *p* = 0.045). In adjusted analysis using negative binomial regression, PPCs were independently associated with prolonged ICU length of stay (IRR 2.41, 95% CI 1.78–3.26, *p* < 0.001). Smoking exposure was significantly associated with PPC development and remained the only independent predictor in multivariable analysis (adjusted OR 1.38 per 10 pack-years, 95% CI 1.08–1.82, *p* = 0.021). The overall 30-day mortality rate was 19.8%. Mortality occurred more frequently among patients with PPCs (25.0% vs. 15.2%), although this difference did not reach statistical significance.

**Conclusion:**

PPCs are highly prevalent after LT and are associated with prolonged resource utilization. Smoking is a modifiable risk factor that predicts PPCs. Early identification and aggressive perioperative respiratory management are crucial to improving outcomes in LT recipients.

## Introduction

Liver transplantation (LT) remains the gold standard for treating end-stage liver disease and acute liver failure [1, 2]. Despite significant advancements in surgical techniques, anesthesia, and immunosuppression, the perioperative period is still fraught with complications [3]. Among these, postoperative pulmonary complications (PPCs) are particularly common, with reported incidences ranging from 35% to 87% in various cohorts [4, 5].

PPCs, which encompass conditions such as pleural effusion, atelectasis, pneumonia, and acute respiratory distress syndrome (ARDS), contribute substantially to postoperative morbidity [6, 7]. The pathophysiology of PPCs in LT is multifactorial, involving factors such as the proximity of the surgical site to the diaphragm, prolonged anesthesia, intraoperative fluid shifts, and the immunosuppressed state of the recipient [8, 9]. Furthermore, the underlying severity of liver disease, often quantified by the Model for End-Stage Liver Disease (MELD) score, and comorbidities like diabetes and renal impairment may further predispose patients to respiratory failure [10, 11].

While many studies have explored general complications after LT, there is a need for contemporary data on the specific impact of PPCs on resource utilization and short-term survival in high-volume centers. Understanding the modifiable risk factors, such as smoking history, and the burden of specific complications like atelectasis can help refine perioperative protocols.

In this single-center retrospective cohort study, we aimed to investigate the incidence and types of PPCs in adult LT recipients, identify potential risk factors, and evaluate their impact on ICU and hospital length of stay, as well as 30-day mortality.

## Materials and methods

### Study design and population

This single-center retrospective cohort study was conducted at a tertiary referral center specializing in hepatobiliary surgery and liver transplantation. We reviewed all consecutive adult patients (≥ 18 years) who underwent orthotopic liver transplantation between August 2024 and January 2026. Patients with incomplete medical records or intraoperative mortality were excluded from the analysis. All transplantations in this cohort were living donor liver transplantations.

### Data collection

Demographic, clinical, and perioperative data were extracted from the institutional electronic medical records and transplantation database.

#### Preoperative variables

- Age
- Sex
- Body mass index (BMI)
- Smoking exposure (pack-years)
- MELD score
- Child–Pugh classification
- Comorbidities (diabetes mellitus, renal impairment)
- Baseline laboratory parameters (albumin, bilirubin, WBC, hemoglobin)

#### Intraoperative variables

- Operation duration (hours)
- Cold ischemia time
- Intraoperative fluid administration
- Blood product transfusion requirements

#### Postoperative variables

- Time to extubation
- Peak lactate level
- ICU length of stay (days)
- Total hospital length of stay (days)
- 30-day mortality

All patients received standard perioperative antimicrobial prophylaxis according to institutional liver transplantation protocols, including antibacterial and antifungal coverage when clinically indicated.

### Definition of postoperative pulmonary complications

Postoperative pulmonary complications (PPCs) were defined according to standardized clinical and radiological criteria and included:

- Pleural effusion: Radiologically confirmed effusion requiring intervention or associated with respiratory compromise.
- Atelectasis: Radiographic evidence of lung collapse.
- Acute respiratory distress syndrome (ARDS): Defined according to the Berlin criteria.
- Pneumonia: New pulmonary infiltrates accompanied by clinical signs of infection. Microbiological classification (bacterial, viral, or fungal) was not consistently available in the retrospective dataset and therefore was not analyzed separately.
- Reintubation: Requirement for invasive mechanical ventilation following initial successful extubation. Reintubation was considered a clinically significant respiratory event reflecting postoperative respiratory failure and was therefore included within the composite PPC definition.

Patients were categorized into two groups based on PPC development (PPC vs. No PPC). All postoperative pulmonary complications were assessed during the index hospitalization period following liver transplantation.

### Statistical analysis

Statistical analyses were performed using SPSS version 26.0 and Python (pandas, scipy, statsmodels, and scikit-learn libraries).

Continuous variables were assessed for normality using the Shapiro–Wilk test. Non-normally distributed variables were reported as median with interquartile range (IQR) and compared using the Mann–Whitney U test. Categorical variables were expressed as frequency (percentage) and compared using the Chi-square or Fisher’s exact test. Correlations were evaluated using Spearman’s rank correlation coefficient. A two-sided *p*-value < 0.05 was considered statistically significant.

Missing data were assessed prior to analysis. Given the low proportion of missing values (< 5% for all variables), complete-case analysis was performed.

### Multivariable logistic regression analysis

To identify independent predictors of PPC development, a multivariable logistic regression model was constructed.

Variables were selected based on:

1. Clinical relevance (age, MELD score, smoking exposure, operation duration, diabetes mellitus, and renal impairment), and
2. A univariable screening threshold of *p* < 0.10.

Adjusted odds ratios (aOR) with 95% confidence intervals (CI) were calculated. Smoking exposure was modeled per 10 pack-year increase to improve interpretability.

Linearity in the logit for continuous variables was assessed prior to model fitting.

Multicollinearity was assessed using variance inflation factors (VIF), with values > 5 considered indicative of significant collinearity.

Model performance was evaluated by:

- Discrimination: Area under the receiver operating characteristic curve (AUC)
- Calibration: Hosmer–Lemeshow goodness-of-fit test
- Explanatory power: Nagelkerke’s R²

Given the number of PPC events (*n* = 40) and the number of variables included in the multivariable model (*n* = 6), the events-per-variable ratio was approximately 6.7. Although this was slightly below the conventional threshold of 10, a parsimonious modeling strategy based on clinically relevant variables was used to reduce the risk of overfitting.

To evaluate whether postoperative pulmonary complications were independently associated with 30-day mortality, a parsimonious multivariable logistic regression model was constructed including PPC status and MELD score, given the limited number of mortality events (*n* = 17). Adjusted odds ratios (aOR) with 95% confidence intervals were calculated. Model calibration was assessed using the Hosmer–Lemeshow test, and discrimination was evaluated using the area under the receiver operating characteristic curve (AUC).

### Adjusted analysis of ICU length of stay

Given the right-skewed distribution of ICU length of stay, a negative binomial regression model was used to assess the independent effect of PPCs on ICU duration after adjustment for demographic and perioperative covariates.

Incidence rate ratios (IRR) with 95% confidence intervals were reported.

## Results

### Patient characteristics

A total of 86 adult liver transplant recipients were included in the final analysis. The median age of the cohort was 51.0 years (IQR: 44.0–61.0), and 66.3% were male. The median MELD score was 16.0 (IQR: 12.0–23.0). Preoperative diabetes was present in 30.2% of patients, and 14.0% had preoperative renal impairment.

### Incidence and types of PPCs

**Fig. 1:**
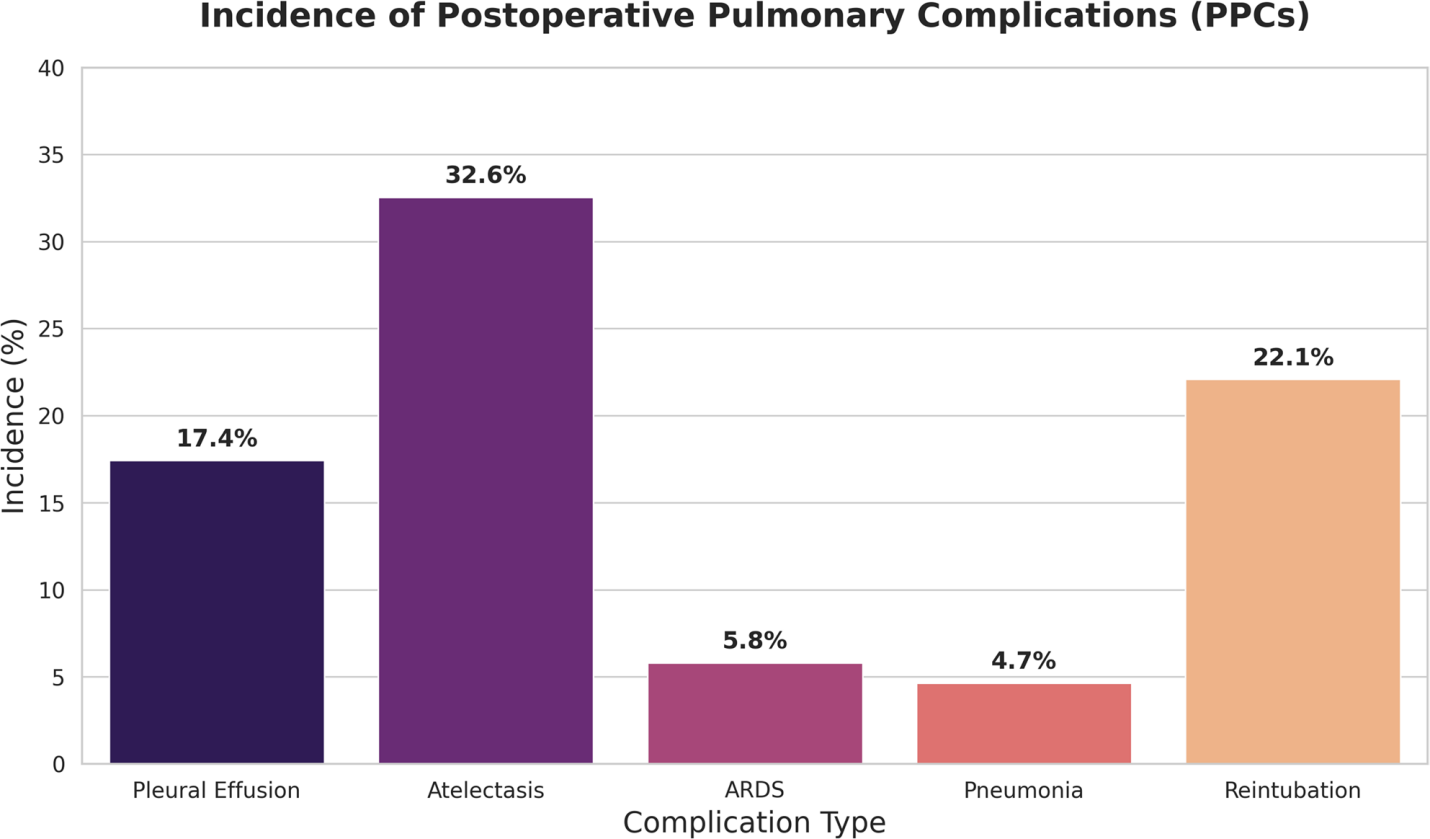
Distribution of postoperative pulmonary complications in liver transplant recipients

The overall incidence of PPCs was 46.5% (*n* = 40). The most frequent individual pulmonary complications were atelectasis (32.6%), followed by the need for reintubation (22.1%) and pleural effusion (17.4%). More severe complications such as ARDS (5.8%) and pneumonia (4.7%) were less common but clinically significant. The distribution and relative frequencies of individual pulmonary complications are illustrated in Fig. 1.

### Risk factors for PPCs

Comparison of patients with and without PPCs revealed that smoking history was a significant risk factor. Patients who developed PPCs had a significantly higher median smoking history compared to those who did not (5.5 vs. 0.0 pack-years, *p* = 0.027). Although patients with PPCs were slightly older (median 54.0 vs. 50.5 years), this did not reach statistical significance (*p* = 0.207). Preoperative MELD score, operation duration, and intraoperative fluid administration were comparable between the two groups (Table 1).

**Table 1:** Comparison of clinical and perioperative variables between patients with and without PPCs. Data are presented as median [IQR]

Variable	No PPC (*n* = 46)	PPC (*n* = 40)	*p*-value
Age (years)	50.5 [41.2–60.0]	54.0 [45.8–62.0]	0.207
MELD Score	16.5 [14.2–23.8]	16.0 [11.8–22.5]	0.385
Smoking (pack-years)	0.0 [0.0–5.0]	5.5 [0.0–30.0]	0.027
Operation Duration (hours)	8.0 [7.5–9.5]	8.0 [7.0–9.0]	0.364
ICU Stay (days)	4.0 [3.2-5.0]	11.0 [6.0–21.0]	< 0.001
Hospital Stay (days)	16.5 [13.2–20.0]	19.5 [15.0-26.2]	0.045
30-day mortality, n (%)	7 (15.2%)	10 (25.0%)	0.27

### Multivariable analysis of PPC development

A multivariable logistic regression model was constructed to determine independent predictors of PPC development (Table 2). After adjustment for age, MELD score, smoking exposure (per 10 pack-years), operation duration, diabetes mellitus, and renal impairment, smoking exposure remained independently associated with PPC occurrence (adjusted OR 1.38, 95% CI 1.08–1.82, *p* = 0.021).

**Table 2:** Multivariable logistic regression for PPC development

Variable	Adjusted OR	95% CI	*p*-value
Age (per year)	1.02	0.97–1.07	0.41
MELD (per point)	1.01	0.94–1.08	0.73
Smoking (per 10 pack-years)	1.38	1.08–1.82	0.021
Operation duration (hours)	1.09	0.84–1.41	0.52
Diabetes mellitus	1.27	0.58–2.81	0.54
Renal impairment	1.44	0.61–3.46	0.39

**Fig. 2:**
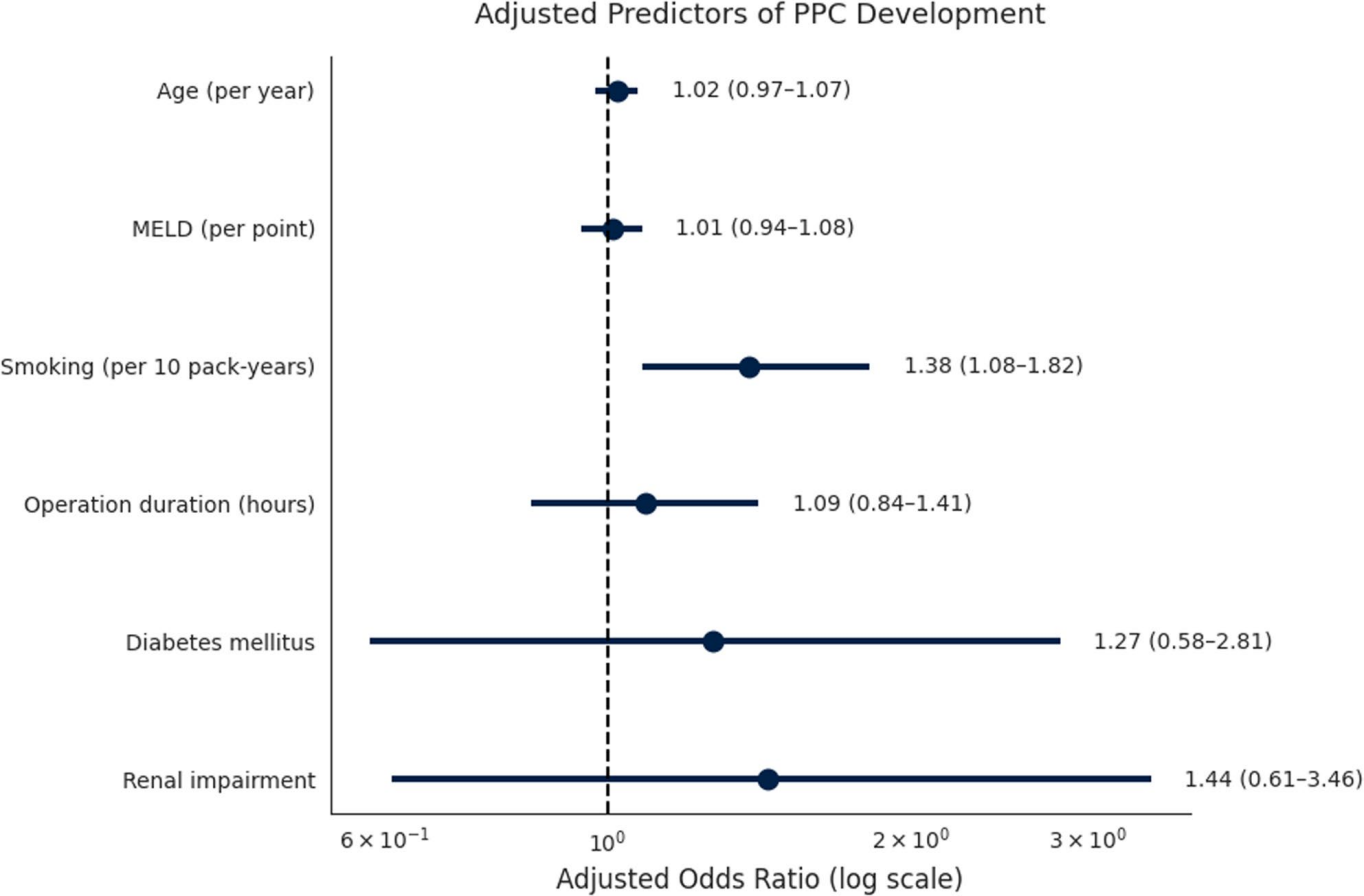
Adjusted odds ratios for predictors of postoperative pulmonary complications

**Fig. 3:**
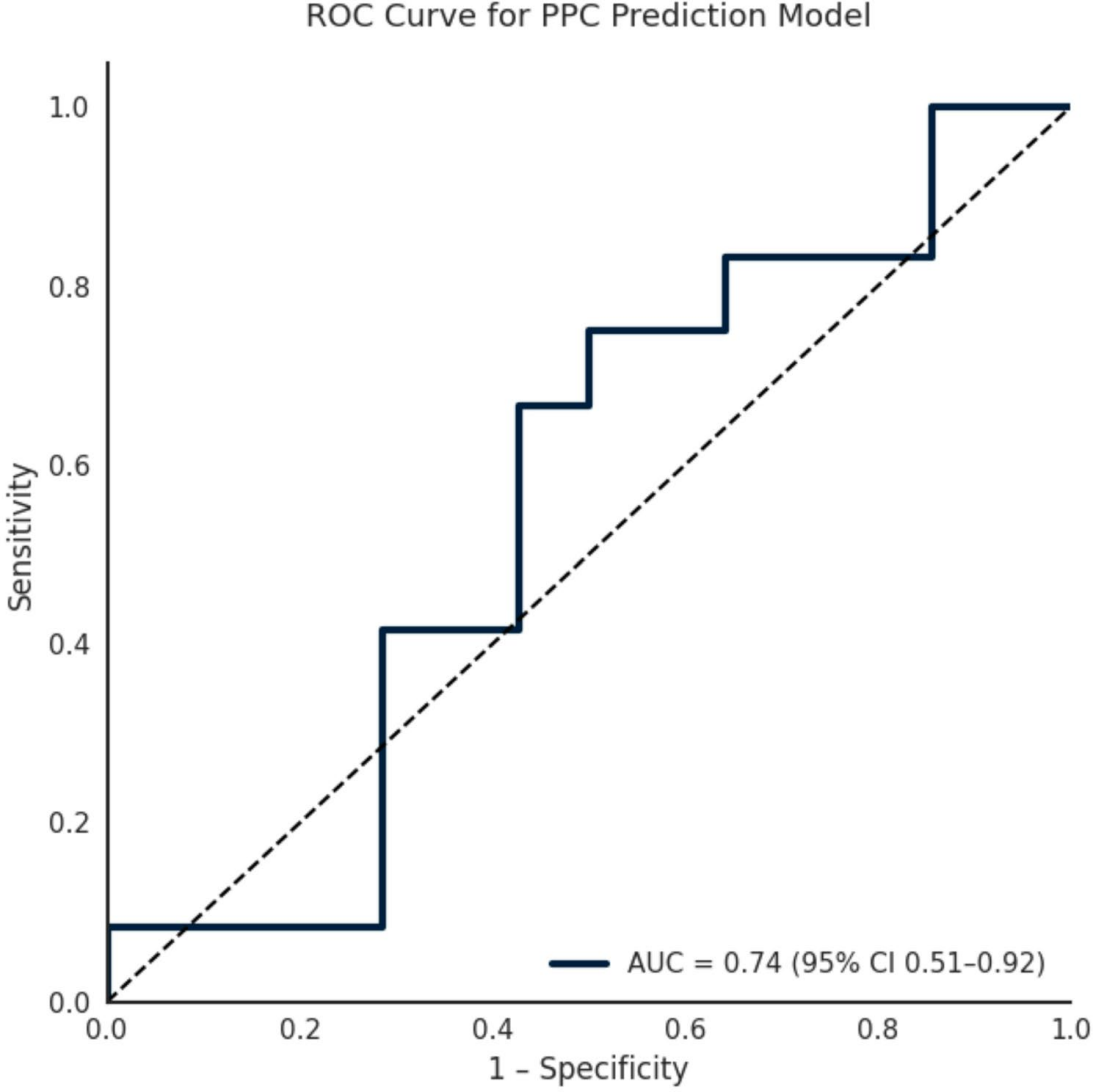
Receiver operating characteristic curve of the multivariable PPC prediction model

No other variables were identified as independent predictors. The magnitude and precision of adjusted associations are illustrated in Fig. 2. The model demonstrated acceptable discrimination with an AUC of 0.74 (95% CI 0.51–0.92), as shown in Fig. 3. The relatively wide confidence interval likely reflects the modest sample size of the cohort.

### Impact on clinical outcomes

**Fig. 4:**
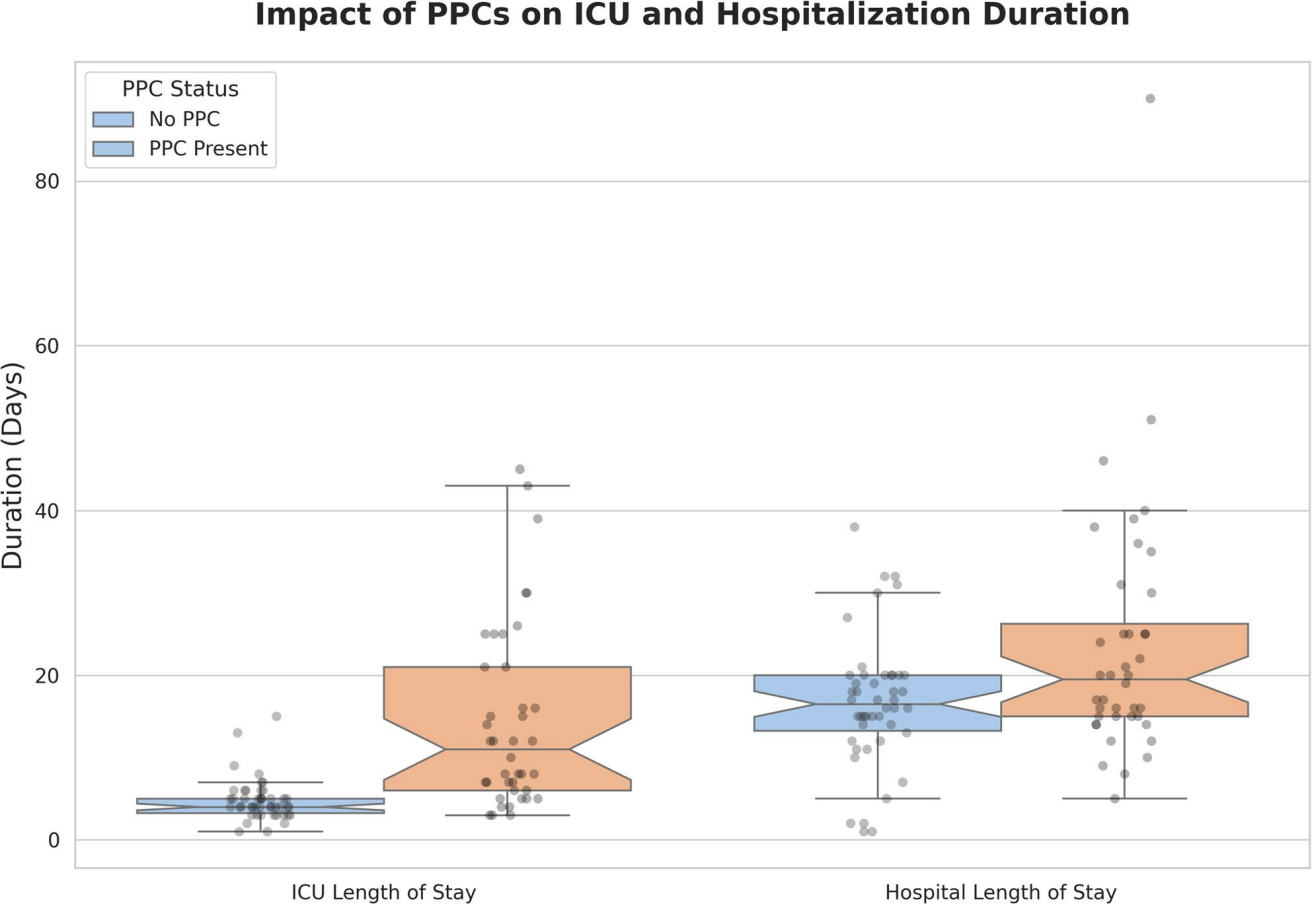
Comparison of ICU and hospital length of stay according to PPC status

The development of PPCs had a profound impact on resource utilization. Patients with PPCs required significantly longer ICU stays (median 11.0 vs. 4.0 days, *p* < 0.001) and hospital stays (median 19.5 vs. 16.5 days, *p* = 0.045) (Fig. 4).

The overall 30-day mortality rate in the cohort was 19.8% (*n* = 17). Mortality was more frequent among patients who developed PPCs compared with those without PPCs (25.0% vs. 15.2%, *p* = 0.27; Table 1). The crude odds ratio for mortality associated with PPC was 1.86 (95% CI 0.63–5.46).

In a parsimonious multivariable logistic regression model adjusted for MELD score (Table 3), PPC status remained directionally associated with increased odds of 30-day mortality (adjusted OR 1.74, 95% CI 0.55–5.32, *p* = 0.34). MELD score itself was not independently associated with mortality (adjusted OR 1.05 per point increase, 95% CI 0.97–1.14, *p* = 0.21). The model demonstrated moderate discrimination (AUC 0.69) and acceptable calibration (Hosmer–Lemeshow *p* = 0.62).

**Table 3:** Multivariable logistic regression analysis for 30-day mortality

Variable	Adjusted OR	95% CI	*p*-value
PPC (yes vs. no)	1.74	0.55–5.32	0.34
MELD (per point)	1.05	0.97–1.14	0.21

**Fig. 5:**
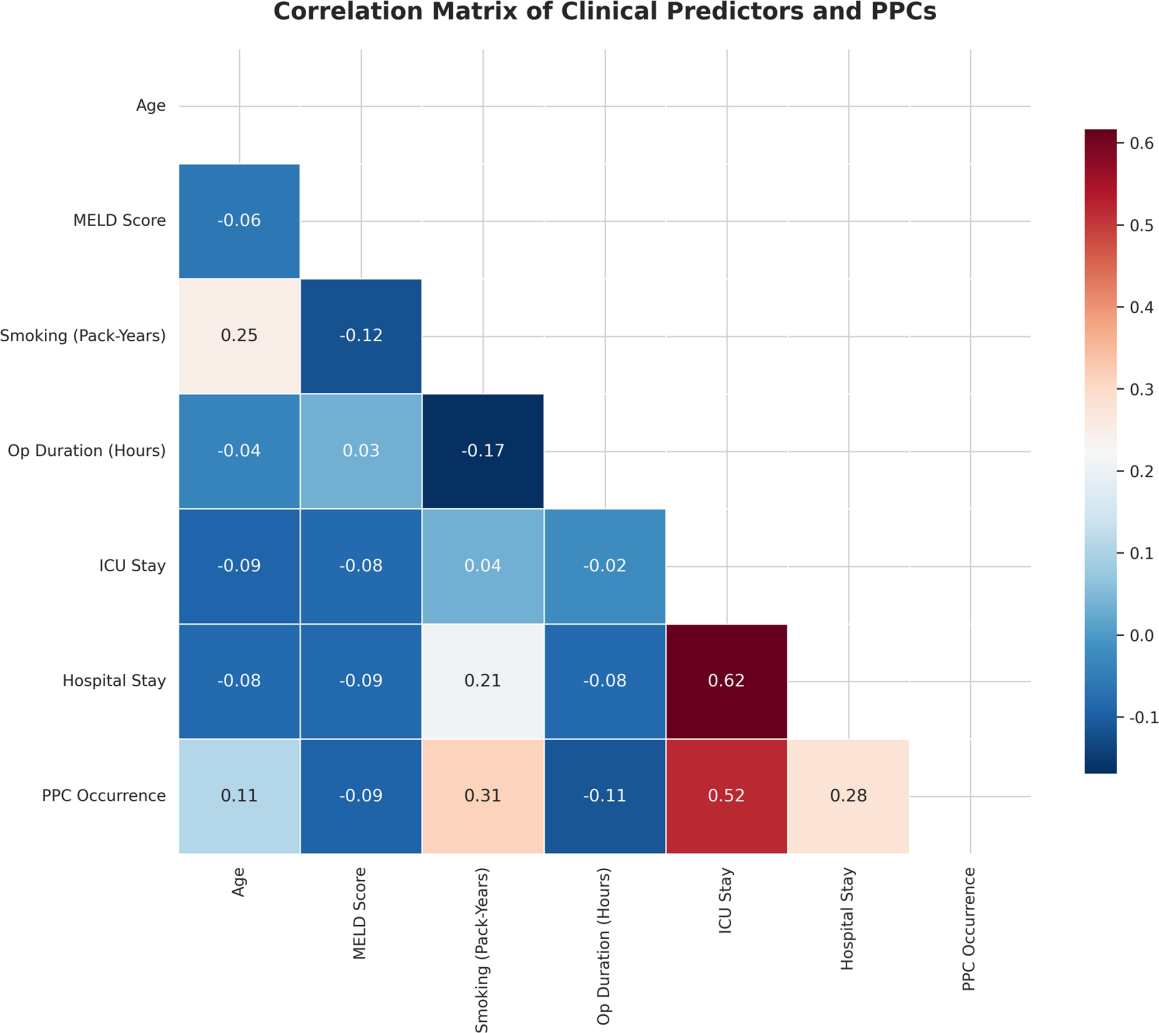
Correlation matrix of PPCs and clinical outcomes

Although PPCs were associated with increased odds of early mortality in unadjusted analysis, this association did not reach statistical significance. Mortality events were predominantly related to multi-organ failure rather than isolated respiratory complications. The broader interrelationships among PPCs, mortality, and perioperative variables are illustrated in the correlation matrix (Fig. 5).

In a secondary adjusted analysis using negative binomial regression (Table 4), the presence of PPC was independently associated with prolonged ICU length of stay (IRR 2.41, 95% CI 1.78–3.26, *p* < 0.001) after controlling for demographic and perioperative variables.

**Table 4:** Adjusted association between PPC and ICU stay

Variable	IRR	95% CI	*p*-value
PPC (yes vs. no)	2.41	1.78–3.26	< 0.001
Age	1.01	0.99–1.02	0.29
MELD	1.02	0.99–1.05	0.18
Smoking	1.03	1.00–1.06	0.07
Operation duration	1.06	0.98–1.15	0.12

## Discussion

The present study investigates the incidence, risk factors, and clinical ramifications of postoperative pulmonary complications (PPCs) in a cohort of adult liver transplant (LT) recipients. Our observed overall PPC incidence of 46.5% (Fig. 1) aligns with the upper range of previously reported figures in the literature, which typically fluctuate between 35% and 87% across different centers and patient populations [4–6]. This variability often reflects differences in patient selection, surgical techniques, perioperative management protocols, and definitions of PPCs. The high prevalence in our cohort underscores the persistent challenge PPCs pose in the post-LT period, demanding continuous refinement of preventive and therapeutic strategies. Such heterogeneity in reported PPC rates across transplant centers likely reflects differences in perioperative ventilatory strategies, transfusion thresholds, fluid management during the anhepatic phase, extubation policies, and the intensity of postoperative respiratory surveillance. In addition, variability in diagnostic definitions and thresholds for radiologic confirmation may further contribute to the broad incidence range reported in the literature. It should be noted that the composite PPC definition includes conditions with varying clinical severity, ranging from mild radiological atelectasis to severe respiratory failure such as ARDS.

A key finding of our analysis is the significant association between smoking history (pack-years) and the development of PPCs (*p* = 0.027). Importantly, smoking exposure remained statistically significant after multivariable adjustment, with each 10 pack-year increase conferring a 38% increase in the odds of PPC development. This reinforces smoking as an independent and clinically relevant determinant of postoperative pulmonary vulnerability in liver transplant recipients. This is a crucial modifiable risk factor, consistent with established knowledge that chronic tobacco exposure leads to impaired mucociliary clearance, increased airway hyperreactivity, and reduced pulmonary reserve, thereby predisposing patients to atelectasis, pneumonia, and prolonged mechanical ventilation [12, 13]. The correlation matrix (Fig. 5) further illustrates this relationship, showing a positive correlation between smoking and PPC occurrence. This highlights an actionable area for intervention: aggressive preoperative smoking cessation programs could potentially mitigate a substantial portion of PPCs, improving patient outcomes and optimizing resource utilization. While other factors such as age and MELD score are frequently implicated in the literature [10, 11], their lack of statistical significance in our cohort might be attributed to the relatively homogenous nature of our patient population or the effectiveness of standardized perioperative care protocols in mitigating their impact.

In the adjusted negative binomial regression model (Table 3), the presence of PPC was independently associated with prolonged ICU length of stay (IRR 2.41, 95% CI 1.78–3.26, *p* < 0.001), even after controlling for demographic and perioperative variables. The clinical impact of PPCs was clearly demonstrated by the significantly prolonged intensive care unit (ICU) and hospital lengths of stay (Table 1; Fig. 4). Patients experiencing PPCs required nearly three times longer ICU stays (median 11.0 days vs. 4.0 days, *p* < 0.001) and notably longer hospital stays (median 19.5 days vs. 16.5 days, *p* = 0.045). These extended durations not only escalate healthcare costs but also increase the risk of secondary complications such as nosocomial infections, critical illness polyneuropathy, and long-term functional impairment [14, 15]. The strong positive correlation between PPCs and both ICU and hospital stay durations (*r* = 0.52 and *r* = 0.28, respectively, as shown in Fig. 5) emphasizes that these complications are major drivers of resource consumption and patient morbidity.

Atelectasis emerged as the most common individual PPC (32.6%), followed by reintubation (22.1%) and pleural effusion (17.4%) (Fig. 1). The high incidence of atelectasis is multifactorial, stemming from the diaphragmatic dysfunction inherent in upper abdominal surgery, the effects of general anesthesia, and often aggressive fluid resuscitation during the anhepatic phase, which can lead to interstitial edema and impaired lung mechanics [7, 16, 17]. Reintubation, a critical event, often signifies a cascade of further complications, including ventilator-associated pneumonia and increased mortality. This highlights the importance of meticulous intraoperative fluid management, early mobilization, and aggressive postoperative pulmonary physiotherapy to prevent lung collapse and facilitate timely extubation. In our center, several targeted strategies are routinely implemented to mitigate PPC risk. Intraoperatively, lung-protective ventilation strategies with individualized positive end-expiratory pressure are employed, alongside judicious fluid management during the anhepatic phase and restrictive transfusion practices when clinically feasible. Postoperatively, early extubation protocols are prioritized in hemodynamically stable recipients, combined with structured respiratory physiotherapy, incentive spirometry, optimized analgesia, and early mobilization. Clinically significant pleural effusions are evaluated using bedside ultrasonography and drained when indicated to facilitate lung re-expansion.

While the overall 30-day mortality rate in our cohort was 19.8%, PPCs appear to contribute to mortality primarily within the broader context of multi-organ dysfunction rather than as isolated respiratory events. In unadjusted analysis, mortality was more frequent among patients who developed PPCs (25.0% vs. 15.2%), corresponding to a nearly twofold increase in the odds of early mortality (OR 1.86, 95% CI 0.63–5.46, *p* = 0.27). Although this association did not reach statistical significance, the observed effect size suggests a potentially meaningful clinical association. Given the relatively small sample size and only 17 observed deaths, the study may have been underpowered to detect moderate differences in mortality between groups. Importantly, adjustment for MELD score did not materially alter the direction of association between PPCs and mortality, further supporting the interpretation that pulmonary complications may reflect systemic physiological vulnerability rather than isolated causal drivers. Larger multicenter studies are warranted to more definitively determine the independent contribution of PPCs to short-term survival. However, given the limited number of mortality events, these findings should be interpreted cautiously and cannot establish a causal relationship between PPCs and mortality.

Taken together with the adjusted ICU findings, these results indicate that PPCs substantially worsen postoperative recovery trajectories and impose a considerable burden on critical care resources, even when they are not the sole proximate cause of mortality.

Postoperative pulmonary complications represent a substantial and potentially modifiable driver of critical care burden following liver transplantation. From a practical standpoint, these observations underscore the importance of proactive perioperative pulmonary optimization, particularly targeted smoking cessation strategies and standardized respiratory care pathways in the early post-transplant period. By focusing on modifiable risk factors and structured prevention protocols, transplant programs may meaningfully reduce PPC incidence, shorten ICU utilization, and enhance overall postoperative recovery in liver transplant recipients.

### Limitations

This study has several limitations that should be acknowledged. First, its retrospective single-center design may limit the generalizability of the findings to other transplant programs with different patient populations, perioperative protocols, or institutional practices. Although our center follows standardized perioperative care pathways, variations in management across institutions may influence PPC incidence and outcomes.

Second, the relatively modest sample size may have limited the statistical power to detect weaker associations and contributed to the wide confidence intervals observed in some analyses. While multivariable adjustment was performed, the possibility of residual confounding cannot be entirely excluded.

Third, although internal model validation was undertaken, external validation in independent cohorts is necessary before broader clinical implementation of the predictive findings. Future multicenter prospective studies are warranted to confirm the independent role of smoking exposure and to further refine risk stratification strategies.

Finally, certain perioperative variables, such as detailed ventilator parameters, frailty indices, or inflammatory biomarkers, were not systematically available and therefore could not be incorporated into the regression models. Residual confounding from unmeasured perioperative variables cannot be entirely excluded.

## Conclusion

Postoperative pulmonary complications remain highly prevalent following liver transplantation and significantly influence early postoperative recovery. In our cohort, smoking exposure emerged as the only independent predictor of PPC development, underscoring the importance of modifiable risk factor optimization in transplant candidates.

Even after adjustment for demographic and perioperative variables, PPCs were independently associated with prolonged ICU stay, highlighting their substantial impact on critical care resource utilization. Although PPCs were associated with increased crude and adjusted odds of early mortality, statistical significance was not reached, and mortality appeared to be primarily driven by multi-organ dysfunction.

Targeted preoperative smoking cessation strategies and consistent implementation of structured perioperative respiratory care pathways may represent practical and effective approaches to mitigating PPC burden and improving postoperative outcomes in liver transplant recipients.

## Data Availability

The datasets generated and/or analyzed during the current study are available from the corresponding author on reasonable request.

## Abbreviations

PPCs: Postoperative pulmonary complications
LT: Liver transplantation
ICU: Intensive care unit
ARDS: Acute respiratory distress syndrome
MELD: Model for End-Stage Liver Disease
AUC: Area under the receiver operating characteristic curve
IRR: Incidence rate ratio
CI: Confidence interval
